# Stable coexistence of two *Caldicellulosiruptor *species in a *de novo *constructed hydrogen-producing co-culture

**DOI:** 10.1186/1475-2859-9-102

**Published:** 2010-12-30

**Authors:** Ahmad A Zeidan, Peter Rådström, Ed WJ van Niel

**Affiliations:** 1 Applied Microbiology, Lund University, Getingevägen 60, SE-222 41 Lund, Sweden

## Abstract

**Background:**

Mixed culture enrichments have been used frequently for biohydrogen production from different feedstock. In spite of the several advantages offered by those cultures, they suffer poor H_2 _yield. Constructing defined co-cultures of known H_2 _producers may offer a better performance than mixed-population enrichments, while overcoming some of the limitations of pure cultures based on synergies among the microorganisms involved.

**Results:**

The extreme thermophiles *Caldicellulosiruptor saccharolyticus *DSM 8903 and *C. kristjanssonii *DSM 12137 were combined in a co-culture for H_2 _production from glucose and xylose in a continuous-flow stirred tank reactor. The co-culture exhibited a remarkable stability over a period of 70 days under carbon-sufficient conditions, with both strains coexisting in the system at steady states of different dilution rates, as revealed by species-specific quantitative PCR assays. The two strains retained their ability to stably coexist in the reactor even when glucose was used as the sole growth-limiting substrate. Furthermore, H_2 _yields on glucose exceeded those of either organism alone under the same conditions, alluding to a synergistic effect of the two strains on H_2 _production. A maximum H_2 _yield of 3.7 mol (mol glucose)^-1 ^was obtained by the co-culture at a dilution rate of 0.06 h^-1^; a higher yield than that reported for any mixed culture to date. A reproducible pattern of population dynamics was observed in the co-culture under both carbon and non-carbon limited conditions, with *C. kristjanssonii *outgrowing *C. saccharolyticus *during the batch start-up phase and prevailing at higher dilution rates. A basic continuous culture model assuming the ability of *C. saccharolyticus *to enhance the growth of *C. kristjanssonii *could mimic the pattern of population dynamics observed experimentally and provide clues to the nature of interaction between the two strains. As a proof, the cell-free growth supernatant of *C. saccharolyticus *was found able to enhance the growth of *C. kristjanssonii *in batch culture through shortening its lag phase and increasing its maximum biomass concentration by ca. 18%.

**Conclusions:**

This study provides experimental evidence on the stable coexistence of two closely related organisms isolated from geographically-distant habitats under continuous operation conditions, with the production of H_2 _at high yields. An interspecies interaction is proposed as the reason behind the remarkable ability of the two *Caldicellulosiruptor *strains to coexist in the system rather than only competing for the growth-limiting substrate.

## Background

In natural ecosystems, mixed microbial populations are the rule rather than the exception. Distinct microbial populations frequently interact with each other in a variety of ways giving rise to many beneficial effects and, therefore, it is not surprising that mixed cultures possess broader metabolic capabilities and show more robustness to environmental fluctuations than individual populations [1]. In biotechnology, continuous enrichment techniques have been used for obtaining stable microbial consortia adapted to continuous culture conditions, which is especially valuable in a continuous-flow commercial process [2]. The use of such enrichments has indeed been proven useful in several applications, such as anaerobic digestion and biopolymer and solvent production [3].

Alternatively, microbial communities can be built, where the diversity and metabolic capabilities of the community can be tuned by careful selection of the individual members for achieving improved or new structures and functions [4]. This approach has been widely adapted in food fermentations [5,6] and successfully employed for lab-scale bioethanol [7-9], acetic acid [10] and lactic acid [11] production. It also provides an essential tool for fundamental studies on the different mechanisms of microbial interactions [12-16]. Although the approach appears interesting, the stability of these artificial communities is remarkably difficult to achieve for various reasons, as discussed by Weibel [4]. To date, there appears to be only few studies that report on the construction of co-cultures consisting of two or more bacterial species that can stably coexist over a long period of time [17-21].

Owing to its high economic value and wide range of applications in the chemical industry, H_2 _has been a subject of increasing interest in recent years. Considerable research efforts have been dedicated to producing H_2 _by biological conversion of biomass via dark fermentation, with a notable emphasis on the use of mixed-culture enrichments [22-25]. The starting culture is usually derived from samples of compost or anaerobic treatment sludge [26] and in spite of the high volumetric H_2 _production rates achieved by these cultures, H_2 _yields usually do not exceed two moles per mole of hexose sugar converted [3]. Since the process is always coupled to biowaste treatment, high yields may not be viewed as essential. However, dark fermentation allows the release of up to 4 moles of H_2 _per mole of hexose sugar [27], and therefore, if H_2 _is to be produced from energy crops, this maximum yield should be aimed for.

Construction of 'designer' H_2_-producing communities can be foreseen as a possible way to overcome some of the issues inherent to the use of undefined consortia, most notably the low H_2 _yields [22,28]. In a previous study, the extreme thermophiles *Caldicellulosiruptor saccharolyticus *and *C. kristjanssonii *have been combined in a H_2_-producing co-culture with the primary aim of improving the rate of glucose consumption during co-fermentation with xylose [28]. A more interesting outcome of this co-culture was its ability to produce H_2 _at a higher yield than that of either organism alone. However, the fermentations were carried out in batch mode and the stability of the co-culture in a continuous system remained questionable. This is particularly important since in practical application chemostat conditions are more likely to be used. In the present work, we demonstrate H_2 _production at high yields in a continuously-stirred tank reactor (CSTR) by a stable co-culture of *C. saccharolyticus *and *C. kristjanssonii *under chemostat conditions. Due to the lack of morphological differences between the two species, a quantitative PCR (qPCR) assay for monitoring the growth of each organism in the co-culture was developed. In addition, a proof of the ability of one or more products of *C. saccharolyticus *cells to enhance the growth of *C. kristjanssonii *could be obtained. The remarkable potential of the two, closely related, strains to stably coexist under nutrient-limited conditions, as could be captured in a simple mathematical model describing the system, is discussed.

## Materials and methods

### Microorganisms

*C. saccharolyticus *DSM 8903 (also, ATCC 43494) and *C. kristjanssonii *strain I77R1B (DSM 12137; ATCC 700853) were purchased from the Deutsche Sammlung von Mikroorganismen und Zellkulturen (DSMZ; Braunschweig, Germany).

### Inoculum development

*C. saccharolyticus *and *C. kristjanssonii *were individually subcultured twice at 70°C in 50 mL modified DSM medium 640 [28] containing the same sugar(s) used in the subsequent fermentation (10 g L^-1^) under N_2 _atmosphere in 250-mL crimped-seal bottles. After the optical density at 620 nm (*OD*_620_) of the second subculture has reached 0.3 - 0.4, it was used to inoculate the bioreactor at a level of 15% (v v^-1^). For initiating the co-culture, an equal volume of each organism was used.

### Bioreactor setup

The organisms were grown in a jacketed, 3-L stirred-tank bioreactor (Applikon, Schiedam, The Netherlands) at 70 ± 1°C. The bioreactor was equipped with an ADI 1025 Bio-Console and an ADI 1010 Bio-Controller (Applikon, Schiedam, The Netherlands). A modified DSM medium 640 was used for all cultivations at a working volume of 1 L. A sterile solution of glucose and/or xylose was added to the medium after sterilization to the required concentration. The medium was continuously sparged with N_2 _gas containing less than 5 ppm O_2 _(AGA Gas AB, Sundbyberg, Sweden) at 100 mL min^-1 ^and stirred at 300 rpm. The pH was maintained automatically at 6.7 ± 0.1 at the operating temperature, using 3 M NaOH as a titrant. The medium was rendered completely anaerobic by the addition of cysteine-HCl at a final concentration of 1 g L^-1 ^prior to inoculation. After an initial growth in batch mode, the bioreactor was fed at the end of the exponential growth phase with a fresh medium, having a similar composition to the start-up medium except for the omission of cysteine-HCL, at 0.04 h^-1^. The feed bottle was continuously sparged with N_2 _to avoid medium displacement by air. After a steady state has been attained, a stepwise increase in the dilution rate (*D*) was carried out. Steady states were assessed after at least 5 volume changes through constant H_2 _and CO_2 _production rates and constant biomass concentration.

### Sampling

Gas samples from the bioreactor's headspace were regularly analyzed for H_2 _and CO_2 _composition. Culture samples were regularly withdrawn for monitoring the biomass concentration. In addition, 10 mL culture samples were aseptically withdrawn and cells were harvested by centrifugation at 5,000 × *g *and 4°C for 12 min, washed twice with 1× phosphate-buffered saline (PBS) by centrifugation and stored at -20°C for genomic DNA extraction. The supernatant was further clarified by passing through a 0.2-μm cellulose acetate filter (Advantec, Tokyo, Japan) and analyzed for sugar consumption and metabolite formation.

### Effect of *C. saccharolyticus *cell-free growth supernatant on *C. kristjanssonii*

The growth supernatant of *C. saccharolyticus *was evaluated for its ability to enhance the growth of *C. kristjanssonii *in batch cultures. Growth supernatants from 50-mL cultures of each organism were collected by centrifugation at 12,000 × *g *and 4°C for 6 min. The supernatant of *C. kristjanssonii *culture was discarded and the cell pellet was retained. The supernatant of *C. saccharolyticus *culture was filtered through a sterile, 0.2-μm-pore-size filter (Sarstedt, Nümbrecht, Germany) to obtain a cell-free spent culture broth in which *C. kristjanssonii *cells were resuspended. All manipulations were carried out inside an anaerobic glove box (Plas Labs Inc., MI, USA) under an atmosphere of N_2_-H_2_-CO_2 _(0.85:0.10:0.05). The 50-mL resuspended culture of *C. kristjanssonii *was combined with either 50 or 100 mL of untreated *C. kristjanssonii *inoculum to inoculate 1 L modified DSM 640 medium, containing 10 g L^-1 ^glucose as a substrate, in the bioreactor. The fermentations were conducted in batch mode under the same conditions described above and biomass concentration was monitored at regular intervals.

### Analytical methods

The concentrations of H_2 _and CO_2 _were determined by gas chromatography, using a dual-channel CP 4900 Micro-GC (Varian, Middelburg, The Netherlands). H_2 _was analyzed on a molecular sieve column (CP-MolSieve 5Å PLOT) with the injector and column temperatures at 80 and 100°C, respectively, whereas CO_2 _was analyzed on a CP-PoraPLOT Q column with the temperature of both the injector and the column at 80°C. The carrier gases for the MolSieve and the PoraPLOT Q columns were N_2 _and He, respectively, at 150 kPa. Each channel was equipped with a micro-machined thermal conductivity detector. Sugars, organic acids and ethanol were separated by high-performance liquid chromatography (HPLC; Waters Corporation, Milford, MA) on an Aminex HPX-87H column (Bio-Rad, Hercules, CA) at 45°C. Sulfuric acid (5 mM) was used as the mobile phase at a flow rate of 0.6 mL min^-1^. The analytes were detected on a refractive index detector (RID-6A; Shimadzu, Tokyo, Japan).

### Biomass determination

A U-1800 spectrophotometer (Hitachi, Tokyo, Japan) was used for regular monitoring of the *OD*_620_. For cell dry weight (CDW) determination, 10 ml of culture were transferred to 15-mL dried, pre-weighed Falcon tubes and centrifuged at 5,000 × *g *for 15 min, washed with deionized H_2_O and dried at 70°C to a constant weight. Enumeration of *C. saccharolyticus *and *C. kristjanssonii *in their pure cultures was performed by direct microscopic count, using a Bürker-pattern counting chamber (Marienfeld, Lauda-Königshofen, Germany).

### DNA extraction

Genomic DNA was extracted from washed, frozen cell pellets using the Easy-DNA Kit (Invitrogen, San Diego, CA), according to the manufacturer's protocol. For complete RNA degradation, DNA preparations in Tris-EDTA buffer (TE buffer; pH 8) were incubated with RNase A (40 μg mL^-1^) for 30 min at 37°C. The concentration and purity of DNA were analyzed spectrophotometrically in an Eppendorf Biophotometer (Hamburg, Germany). Aliquots of DNA in TE buffer were stored at -20°C until use.

### Real-time PCR assays

qPCR assays were developed for quantifying *C. saccharolyticus *and *C. kristjanssonii *in their co-culture. For this, specific oligonucleotide primer pairs based on differences in the 16S rRNA gene sequence of the two species, as retrieved from the GenBank Sequence Database http://www.ncbi.nih.gov/Genbank/, were designed (Table 1). The gene sequences were compared for differences using the ClustalW2 multiple sequence alignment program http://www.ebi.ac.uk/Tools/clustalw2/. Real-time PCR amplification and detection were performed in the LightCycler 2.0 Instrument (Roche Diagnostics, Mannheim, Germany). The PCR mixture (20 μl) contained 1× *Tth *PCR buffer, 1 U *Tth *DNA polymerase (both, Roche Diagnostics), 1.5 mM MgCl_2_, 0.2 mM dNTPs (both, Fermentas, St. Leon-Rot, Germany), 1× SYBR Green I solution (Roche Diagnostics) and 4 μl DNA template solution, in addition to the forward and reverse primers. The primer concentration in the PCR mixture was 0.125 μM (each) for *C. saccharolyticus*-specific assay and 0.5 μM (each) for *C. kristjanssonii*-specific assay. Blanks containing sterile MilliQ water (Millipore; Billerica, MA, USA) instead of the DNA templates and negative controls containing DNA template of the non-targeted species were included in each run. The LightCycler amplification protocol started with an initial denaturation at 95°C for 60 s, followed by 45 cycles of denaturation at 95°C for 0 s, annealing and fluorescence acquisition at 63°C for 5 s, and elongation at 72°C for 25 s. The specificity of the primers was validated so that the quantitative signals detected for the target strain were not affected by the presence of genomic DNA of the other strain. For that, a melting-curve analysis, consisting of heating at 95°C for 0 s and 50°C for 15 s followed by an increase in temperature by 0.2°C/s up to 90°C, was performed. In addition, the PCR products were further confirmed by agarose-gel electrophoresis.

**Table 1:** Real-time PCR primers for quantification of *C. saccharolyticus* and *C. kristjanssonii* in their co-culture.

Target	Primer	Sequence	Product size (bp)
*C. saccharolyticus*	S_572F	GGTGCGTAGGCGGCTATGCG	448
	S_1019R	CCCACCCTTTCGGGCAGGTC	
*C. kristjanssonii*	K_612F	GGAGCGCTCAAGACTGCCGG	317
	Ks_928R	TCCACCGCTTGTGCGGGCC	

Quantification was achieved by determining the crossing point (Cp) of the sample - that is, the fractional cycle number that corresponds to the maximum of the second derivative of the amplification curve - and comparing to standards. For preparing the standards, pure cultures of *C. saccharolyticus *and *C. kristjanssonii *were individually grown on glucose in 3-L bioreactors under the same conditions described above, albeit in batch mode. Culture samples of each organism were collected at different time intervals for cell count and genomic DNA extraction. A first standard curve between DNA concentration and cell number was plotted to validate the DNA extraction protocol and the capacity of the extraction kit. DNA preparations from samples of varying cell numbers were then used, after appropriate dilution, as templates for qPCR. The mean Cp of two independent real-time PCR replicates was plotted against the logarithm of the corresponding cell count to obtain a second standard curve. For quantitative determination *C. saccharolyticus *and *C. kristjanssonii *in different samples of their co-culture, the second standard was regenerated for each species with every run in the LightCycler to overcome any variability in the amplification efficiency.

### Co-culture model

A mathematical model was built to simulate the stable co-existence of *C. saccharolyticus *(organism 1) and *C. kristjanssonii *(organism 2) in the co-culture, with glucose as the main carbon and energy source. The model is based on the general mass balance equations and the proposed release of a compound (*E*) by *C. saccharolyticus *with the ability to enhance biomass yield and the maximum specific growth rate of *C. kristjanssonii*. The continuous co-culture system can be described by three relevant differential equations (growth of *C. saccharolyticus*, growth of *C. kristjanssonii *and glucose consumption, respectively):

(1) $$\frac{dx_{1}}{dt}=(\mu_{1}-D)\;\;x_{1}$$

(2) $$\frac{dx_{2}}{dt}=(\mu_{2}-D)\;\;x_{2}$$

(3) $$\frac{ds}{dt}=D\;\;(s_{0}-s)\;\;-\frac{1}{Y_{sx_{1}}}\mu_{1}\;x_{1}-\frac{1}{Y_{sx_{2}}}\mu_{2}\;x_{2}$$

where *x*_1 _is the biomass concentration of *C. saccharolyticus *(gCDW L^-1^), *x*_2 _is the biomass concentration of *C. kristjanssonii *(gCDW L^-1^), *s*_0 _is the glucose concentration in the feed (mmol L^-1^), *s *is the residual glucose concentration (mmol L^-1^), and *t *is the fermentation time (h). The biomass yield coefficient $Y_{sx_{1}}$ [gCDW (mmol glucose^-1^)], is considered to be constant, whereas $Y_{sx_{2}}$ and the growth rates (*μ*_1 _and *μ*_2_) are each a function of several variables as explained below.

The growth rate of *C. saccharolyticus *and *C. kristjanssonii *can be described by Monod equations:

(4) $$\mu_{1}=\mu_{\max1\;}\frac{s}{K_{s_{1}}+s}$$

(5) $$\mu_{2}=\mu_{\max2\;}\frac{s}{K_{s_{2}}+s}$$

in which *μ*_max1 _and *μ*_max2 _are the maximum specific growth rates (h^-1^) of *C. saccharolyticus *and *C. kristjanssonii*, respectively, and $K_{s_{1}}$ and $K_{s_{2}}$ are the Monod constants for glucose (mmol L^-1^) for *C. saccharolyticus *and *C. kristjanssonii*, respectively.

The relationship between the concentration of the proposed excreted product (*E*) and *C. saccharolyticus *biomass concentration is as follows:

(6) $$E=\kappa x_{1}$$

with κ as the specific yield factor (mmol gCDW^-1^). The increase in the maximum specific growth rate and biomass yield of *C. kristjanssonii *by the effect of compound *E *can be described as:

(7) $$\mu_{\max2}=\mu_{\max2}^{0}-(\mu_{\max2}^{'}-\mu_{\max2}^{0})\;\frac{E}{E_{C}}$$

(8) $$Y_{sx_{2}}=Y_{sx_{2}}^{0}-(Y_{sx_{2}}^{'}-Y_{sx_{2}}^{0})\;\frac{E}{E_{C}}$$

with $\mu_{\max2}^{0}$ and $\mu_{\max2}^{'}$ as the maximum specific growth rates in the absence of compound *E *and in its presence at the critical concentration (*E*_C_), respectively. Likewise, $Y_{sx_{2}}^{0}$ and $Y_{sx_{2}}^{'}$ are the biomass yield factors in the absence of compound *E *and in its presence at the critical concentration, respectively.

Values of the different parameters included in the model are listed in Additional file 1 (Table S1). The set of differential equations was solved using Matlab (R2009a; The MathWorks, Inc.).

## Results

### Hydrogen production and stability of the co-culture under non-carbon limitation

*C. saccharolyticus *and *C. kristjanssonii *were grown together in a CSTR at various dilution rates, ranging from 0.04 to 0.3 h^-1^, over a period of 70 days. The total sugar concentration in the feed was 10 g L^-1 ^(glucose and xylose; 1:1). At least 8 volume changes were required to attain a steady state at lower dilution rates, while up to 20 volume changes were required at the higher ones. Neither glucose nor xylose in the feed was completely consumed at any of the dilution rates tested, suggesting that the co-culture was not carbon limited (Table 2). The residual sugar concentration increased with the dilution rate, and a similar trend was observed for biomass concentration. The specific xylose consumption rate was considerably higher than that of glucose at all dilution rates (see Table S2.1 in Additional file 2). The main metabolic end products of glucose and xylose fermentation were H_2_, CO_2 _and acetate, whereas lactate was formed in minor quantities. Complete carbon and electron recoveries could be obtained (see Table S2.2 in Additional file 2).

**Table 2:** H_2_ production and biomass formation by the co-culture on a mixture of glucose (5 g L^-1^; 27.8 mM) and xylose (5 g L^-1^; 33.3 mM) at steady states of different dilution rates (yeast extract, 1 g L^-1^; pH 6.7; 70°C).

***D *(h**^**-1**^**)**	$Y_{{\text{H}}_{\text{2}}}$**[mol (mol C6)**^**-1**^**]**	$Q_{{\text{H}}_{\text{2}}}$**(mmol L**^**-1 **^**h**^**-1**^**)**	$q_{{\text{H}}_{2}}$**(mmol gCDW**^**-1 **^**h**^**-1**^**)**	**Biomass concentration (gCDW L**^**-1**^**)**	***Y***_**sx **_**[gCDW (mol C6)**^**-1**^**]**	**Residual sugar (mM)**	
						**Glucose**	**Xylose**
0.04	3.5	3.8	12.7	0.30	11.1	18.4	8.4
0.06	3.6	4.3	14.2	0.30	14.9	18.9	12.0
0.08	3.5	6.2	15.5	0.40	18.0	19.0	9.3
0.12	3.1	8.3	15.0	0.55	24.4	21.4	12.1
0.15	2.9	10.3	18.4	0.56	23.6	21.0	12.4
0.2	2.8	11.0	18.3	0.60	30.7	22.7	15.2
0.25	2.9	11.6	21.0	0.55	34.6	24.2	17.8
0.3	2.5	11.6	21.0	0.55	36.1	23.9	19.0

Both the volumetric and specific H_2 _production rates ($Q_{{\text{H}}_{\text{2}}}$ and $q_{{\text{H}}_{2}}$, respectively) markedly increased with the dilution rate (Table 2). The maximum volumetric hydrogen production rate obtained was 11.6 mmol L^-1 ^h^-1 ^at 0.25 and 0.3 h^-1^. On the other hand, H_2 _yield ($Y_{{\text{H}}_{\text{2}}}$) was inversely proportional to the dilution rate, with the maximum of 3.6 mol H_2 _per mol of hexose equivalent obtained at 0.06 h^-1 ^(Table 2). This yield represents 90% of the maximum theoretical yield that can be obtained from glucose during dark fermentative hydrogen production. The biomass yield (*Y*_sx_) increased sharply at *D *> 0.15 h^-1 ^(Table 2), which could be due to the accumulation of a storage material, e.g., glycogen.

**Figure 1:**
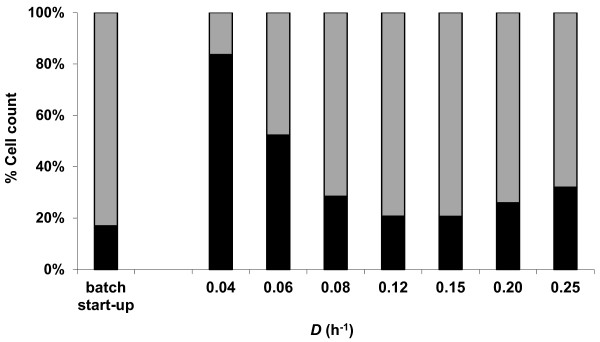
**Monitoring the population dynamics in the co-culture by qPCR**. Relative abundance of *C. saccharolyticus *(black bars) and *C. kristjanssonii *(grey bars) in the co-culture at steady states of different dilution rates during growth on glucose and xylose (10 g L^-1^; 1:1) at pH 6.7 and 70°C. Values expressed are the means of real-time PCR duplicates that varied by less than 10%.

qPCR assays were used to assess the ability of *C. saccharolyticus *and *C. kristjanssonii *to coexist under steady-state conditions and to monitor the population dynamics during the continuous operation of the reactor. The results showed that, in spite of the variation in population dynamics at different steady states, the two organisms could stably coexist over the whole range of dilution rates tested (Figure 1). *C. kristjanssonii *outgrew *C. saccharolyticus *by the end of the batch start-up phase, and at steady states of *D *> 0.06 h^-1^. On the other hand, *C. saccharolyticus *prevailed at the lowest *D*, i.e., 0.04 h^-1^. The specificity of the designed primers was confirmed throughout the assays by melting-curve analysis, where only one amplicon with the expected melting temperature was detected. Additionally, the PCR products were analyzed by agarose-gel electrophoresis, where only one band of the expected size could be detected (data not shown).

### Carbon limitation with two sugars

In order to study the effect of carbon limitation on H_2 _production and the possibility of coexistence of the two species, the total sugar concentration in the feed was reduced to 4 g L^-1 ^(glucose and xylose; 1:1). Based on the co-culture performance under non-carbon limitation, only two dilution rates were chosen as a platform for evaluating H_2 _production efficiency and analyzing the population dynamics, i.e., 0.06 and 0.15 h^-1^. No residual glucose or xylose could be detected at steady state of *D *= 0.06 h^-1^, confirming that the co-culture was carbon limited (Table 3). H_2 _yield decreased with the dilution rate and was comparable to that obtained under non-carbon limitation at the two steady states evaluated. H_2 _production rate followed an opposite trend, since it increased with the dilution rate, as was seen under non-carbon limitation. Product yields and sugar consumption and metabolite formation rates as well as carbon and electron recoveries at the two dilution rates are detailed in Additional file 2 (Tables S3.1 and S3.2).

**Table 3:** Performance of the co-culture in a carbon-limited chemostat at steady states of two different dilution rates with glucose and xylose (4 g L^-1^; 1:1) as the growth limiting substrates (yeast extract, 1 g L^-1^; pH 6.7; 70°C).

***D *(h**^**-1**^**)**	$Y_{{\text{H}}_{\text{2}}}$**[mol (mol C6)**^**-1**^**]**	$Q_{{\text{H}}_{\text{2}}}$**(mmol L**^**-1 **^**h**^**-1**^**)**	$q_{{\text{H}}_{2}}$**(mmol gCDW**^**-1 **^**h**^**-1**^**)**	**Biomass concentration (gCDW L**^**-1**^**)**	***Y***_**sx **_**[gCDW (mol C6)**^**-1**^**]**	**Residual sugar (mM)**	
						**Glucose**	**Xylose**
0.06	3.6 ± 0.1	4.5 ± 0.2	10.2 ± 0.3	0.44 ± 0.03	21.0 ± 0.9	0	0
0.15	3.2 ± 0	8.6 ± 0.4	24.7 ± 1.1	0.35 ± 0.03	19.4 ± 1.0	1.7 ± 0.1	1.4 ± 0.1

Values are means of two independent replicates ± standard deviation.

**Figure 2:**
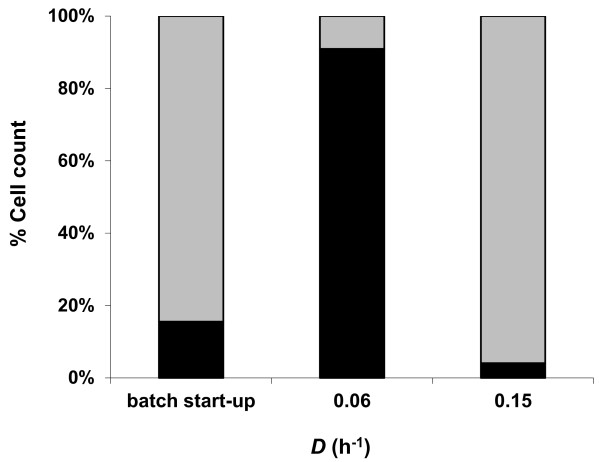
**Relative abundance of *C. saccharolyticus *(black bars) and *C. kristjanssonii *(grey bars) in the co-culture on glucose and xylose (4 g L^-1^; 1:1) at pH 6.7 and 70°C at the end of the batch start-up phase and during steady state growth at two different dilution rates**. Values expressed are the means of real-time PCR duplicates that varied by less than 10%. The figure is a representative of the population dynamics in two independent chemostat cultures.

The steady-state residual sugar concentration increased by increasing *D *(Table 3), while the overall steady-state biomass concentration decreased from 0.44 ± 0.03 gCDW L^-1 ^at 0.06 h^-1 ^to 0.35 ± 0.03 gCDW L^-1 ^at 0.15 h^-1^, which might indicate that the culture was approaching its critical *D*. Although the co-culture was carbon limited, neither *C. saccharolyticus *nor *C. kristjanssonii *washed out, as revealed by qPCR analysis (Figure 2). *C. kristjanssonii *cells constituted around 85% of the total population at the onset of starting the continuous cultivation mode, whereas its steady-state relative cell count went below 10% at *D *= 0.06 h^-1^. The organism, however, restored its predominance when *D *was increased to 0.15 h^-1^, depicting its behaviour under non-carbon limitation.

### Competition for one growth-limiting substrate

The ability of *C. saccharolyticus *and *C. kristjanssonii *to stably coexist under chemostat conditions was further challenged by growing the co-culture on only one sugar, as the growth-limiting substrate, at a time. Glucose was added to the feed at a concentration of 4 g L^-1^, and the performance of the co-culture was also compared to that of the individual species under the same conditions. Glucose was almost completely depleted at both dilution rates by the co-culture and by the pure culture of *C. kristjanssonii *(Table 4). In pure culture of *C. saccharolyticus*, a residual steady-state glucose concentration of 8.6 ± 0.6 mM was detected in the effluent by increasing *D *from 0.06 to 0.15 h^-1^, with a concomitant decrease in biomass concentration. The H_2 _yields obtained by the co-culture were higher than the corresponding yields obtained by either species in pure culture at both dilution rates. The highest volumetric H_2 _production rate was achieved in *C. kristjanssonii *pure culture and in its co-culture with *C. saccharolyticus *at *D *= 0.15 h^-1^, whereas the highest specific H_2 _production rate was solely achieved by the former culture at the same *D *(Table 4). A thorough comparison of product distribution in the pure culture of each strain and in the co-culture as well as carbon and electron recoveries can be found in Additional file 2 (Tables S4.1 and S4.2).

**Table 4:** Comparison of steady-state H_2_ and biomass production by *C. saccharolyticus*, *C. kristjanssonii* and their co-culture in glucose-limited chemostat cultivations at two different dilution rates (yeast extract, 1 g L^-1^; pH 6.7; 70°C).

**Organism(s)**	***D *(h**^**-1**^**)**	$Y_{{\text{H}}_{\text{2}}}$**[mol (mol C6)**^**-1**^**]**	$Q_{{\text{H}}_{\text{2}}}$**(mmol L**^**-1 **^**h**^**-1**^**)**	$q_{{\text{H}}_{2}}$**(mmol gCDW**^**-1 **^**h**^**-1**^**)**	**Biomass concentration (gCDW L**^**-1**^**)**	***Y***_**sx **_**[gCDW (mol C6)**^**-1**^**]**	**Residual glucose (mM)**
*C. saccharolyticus*	0.06	3.5 ± 0.1	4.9 ± 0.14	13.2 ± 0.1	0.38 ± 0.01	15.8 ± 0.2	0
	0.15	3.1 ± 0.2	7 ± 0.3	24.4 ± 1	0.29 ± 0.03	19.3 ± 1.1	8.6 ± 0.6
*C. kristjanssonii*	0.06	3.5 ± 0.1	4.8 ± 0.1	17.8 ± 0.5	0.27 ± 0.01	11.7 ± 0.0	0
	0.15	3.0 ± 0.1	10.3 ± 0.16	34.6 ± 1.6	0.3 ± 0.02	13.0 ± 0.1	0.2 ± 0.0
Co-culture	0.06	3.7 ± 0.0	4.8 ± 0.2	14.8 ± 0.8	0.33 ± 0.03	15.4 ± 0.8	0
	0.15	3.5 ± 0.0	10.4 ± 0.14	21.4 ± 0.8	0.49 ± 0.01	24.6 ± 0.8	0

Values are means of two independent replicates ± standard deviation.

**Figure 3:**
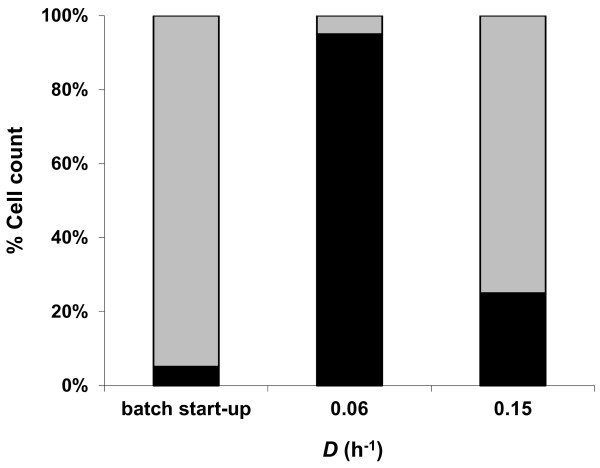
**Relative abundance of *C. saccharolyticus *(black bars) and *C. kristjanssonii *(grey bars) in the co-culture in a glucose-limited chemostat culture at pH 6.7 and 70°C at the end of the batch start-up phase and during steady state growth at two different dilution rates**. Values expressed are the means of real-time PCR duplicates that did vary by less than 10%. The figure is a representative of the population dynamics in two independent chemostat cultures.

Interestingly, both organisms coexisted in the system despite the culture being truly limited for one carbon source, i.e., glucose. *C. kristjanssonii *was the predominant species in the co-culture during the batch start-up phase and at the higher *D *(Figure 3), underlining a similar pattern to that obtained during co-fermentation of glucose and xylose. It is noteworthy that a similar pattern of population dynamics was also obtained with an opposite sequence of dilution rates of this experiment, where a steady state was achieved first at *D *= 0.15 h^-1 ^and then at *D *= 0.05 h^-1 ^(data not shown).

Unlike glucose, xylose could not be completely utilized by the co-culture at either dilution rate when it was used as the sole sugar in the feed at 4 g L^-1 ^(26.7 mM; Table 5). The reason for that is unclear since xylose appears to be the preferable sugar during co-fermentation with glucose (cf., Table 2). By increasing *D*, more xylose was consumed and steady-state biomass concentration has almost doubled. H_2 _yield of the co-culture on xylose did not appear to vary with the dilution rate, and was lower than that obtained on glucose. Specific H_2 _production rate, however, followed an opposite trend, since it was significantly higher on xylose at both dilution rates. The same applies for the specific production rates of all other fermentation products (see Additional file 2: Table S5.1), which could be attributed to the low biomass concentration obtained on xylose.

**Table 5:** Steady-state H_2_ and biomass production by the co-culture on xylose (4 g L^-1^) in the presence of yeast extract (1 g L^-1^) at two different dilution rates (pH 6.7; 70°C).

***D *(h**^**-1**^**)**	$Y_{{\text{H}}_{\text{2}}}$**[mol (mol C6)**^**-1**^**]**	$Q_{{\text{H}}_{\text{2}}}$**(mmol L**^**-1 **^**h**^**-1**^**)**	$q_{{\text{H}}_{2}}$**(mmol gCDW**^**-1 **^**h**^**-1**^**)**	**Biomass concentration (gCDW L**^**-1**^**)**	***Y***_**sx **_**[gCDW (mol C6)**^**-1**^**]**	**Residual xylose (mM)**
0.06	2.7 ± 0.1	3.0 ± 0.2	22.6 ± 4.1	0.13 ± 0.04	7.4 ± 1.7	6.9 ± 0.9
0.15	2.7 ± 0.0	8.5 ± 0.2	33.0 ± 8.9	0.27 ± 0.08	13.0 ± 3.7	3.7 ± 1.4

Values are means of two independent replicates ± standard deviation.

**Figure 4:**
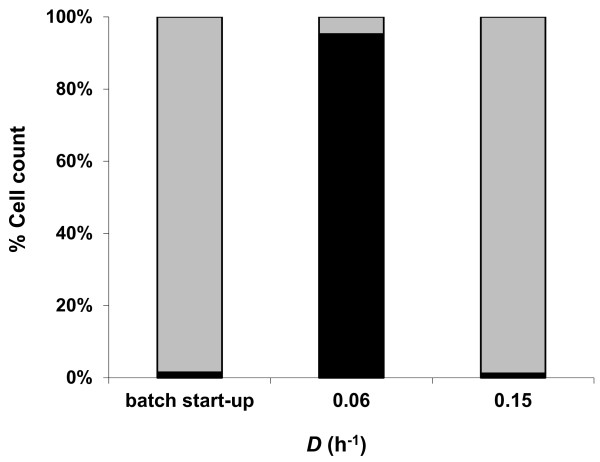
**Relative abundance of *C. saccharolyticus *(black bars) and *C. kristjanssonii *(grey bars) in the co-culture on xylose (4 g L^-1^) at pH 6.7 and 70°C at the end of the batch start-up phase and during steady state growth at two different dilution rates**. Values expressed are the means of real-time PCR duplicates that varied by less than 10%. The figure is a representative of the population dynamics in two independent chemostat cultures.

The remarkable ability of *C. saccharolyticus *and *C. kristjanssonii *to stably co-exist was retained during growth on xylose. Although *C. saccharolyticus *cells constituted less than 5% of the total population in the co-culture during the batch start-up phase, the organism could outgrow *C. kristjanssonii *in the co-culture at steady state of *D *= 0.06 h^-1 ^(Figure 4), as was seen on glucose and on glucose and xylose mixture. The relative abundance of *C. kristjanssonii *in the co-culture on xylose at the end of the batch start-up phase and at both dilution rates matched the behaviour of the organism in all other fermentations.

### Capturing the population dynamics in the co-culture *in silico*

**Figure 5:**
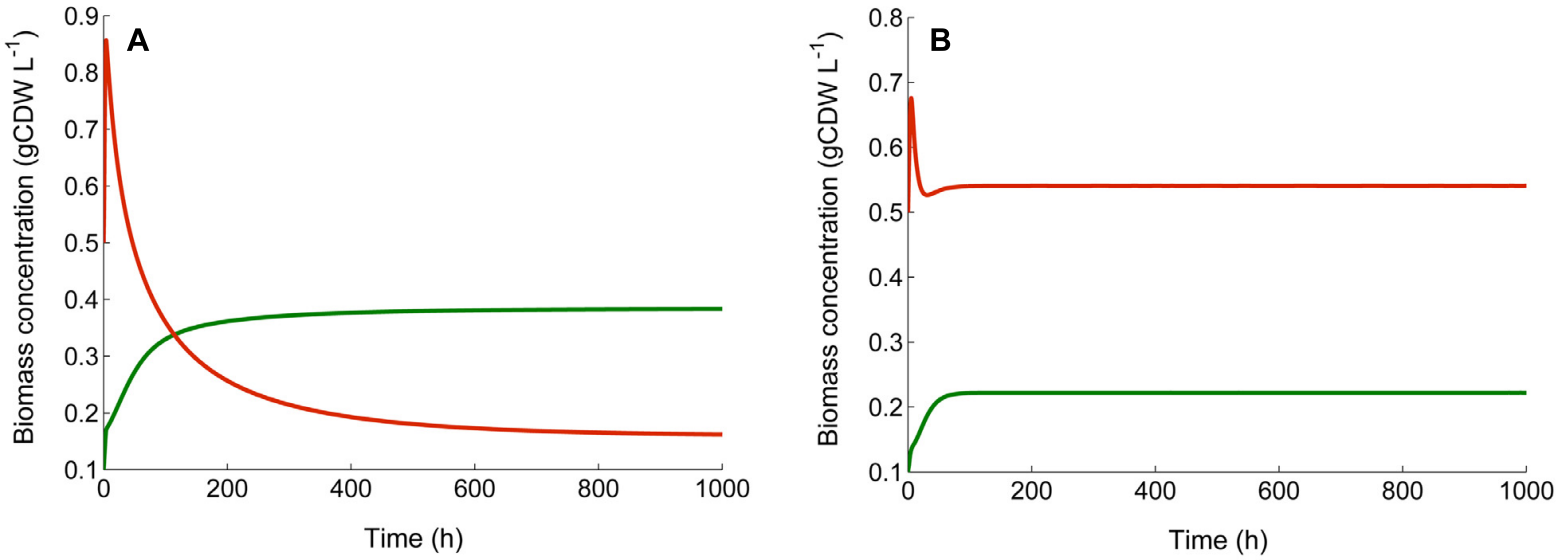
***In silico *glucose-limited chemostat cultivations**. Simulation of the population dynamics in the co-culture on glucose (4 g L^-1^) in chemostat cultures at *D *of 0.06 h^-1 ^(A), and *D *of 0.15 h^-1 ^(B), using the developed mathematical model. Green lines represent *C. saccharolyticus *and red lines represent *C. kristjanssonii*.

It was previously reported that *C. kristjanssonii *exhibits a lower maximum specific growth rate than that of *C. saccharolyticus *during batch growth on glucose and xylose [28]. The consistent predominance of *C. kristjanssonii *in the co-culture during batch growth and at higher *D *under all conditions tested in this study alluded to the possibility of the growth of the organism being enhanced by one or more compounds released by *C. saccharolyticus*. Such an interaction could be a possible reason behind the stable coexistence of the two strains even in the presence of only one growth-limiting substrate. For testing this hypothesis, a mathematical model based on ordinary differential equations for a continuous culture system was extended with equations describing the enhancement of biomass yield and the maximum specific growth rate of *C. kristjanssonii *by a proposed compound released by *C. saccharolyticus *(see Materials and Methods section). Indeed, the model was able to mimic the population dynamics in the co-culture on glucose (Figure 5), provided that well-estimated values of the parameters were used (see Additional file 1: Table S1). Testing the model, it revealed that enhancing the maximum specific growth rate was the most profound effect, i.e., in the absence of any enhancing effect on this variable *C. kristjanssonii *would have washed out at all *D*'s (data not shown). A lack of the stimulatory effect on the biomass yield of *C. kristjanssonii *would have made *C. saccharolyticus *dominant at all times, but the former would have remained in the system (data not shown). According to the model, at higher *D *(i.e., 0.15 h^-1^) a true steady state is attained after ca. 15 volume changes, whereas at a lower *D *(i.e., 0.06 h^-1^) it will be reached after ca. 50 volume changes. In practice, however, sampling after 18 and 9 volume changes from the cultures at a *D *of 0.15 and 0.06 h^-1^, respectively, indicated that a steady state has been attained. This difference between the experimentally determined and model-predicted number of volume changes could be a result of non-optimum values of any of the model parameters for which no experimental data were available (Additional file 1). Extending the *in silico *simulation beyond 1000 h revealed that *C. kristjanssonii *never washed out at either *D *(data not shown).

### Effect of *C. saccharolyticus *culture supernatant on growth of *C. kristjanssonii*

**Figure 6:**
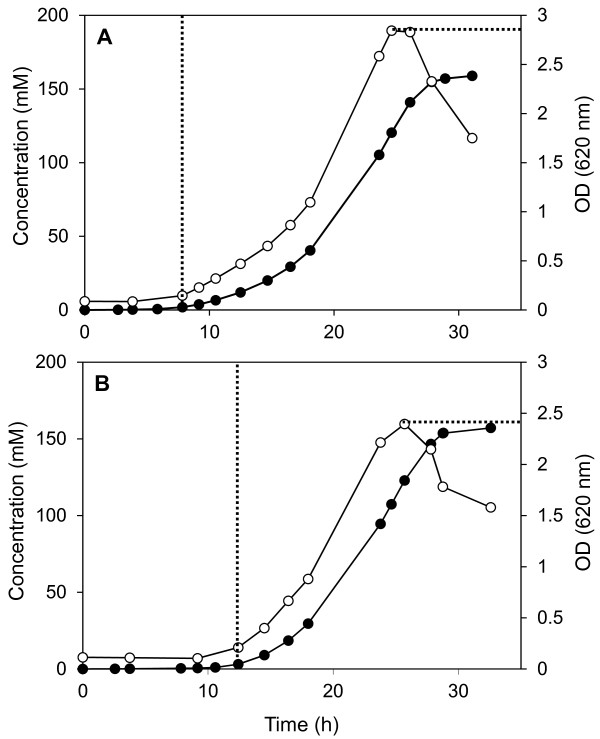
**Effect of *C. saccharolyticus *growth supernatant on *C. kristjanssonii***. Comparison of log phase and maximum biomass concentration of *C. kristjanssonii *on glucose (10 g L^-1^) at pH 6.7 and 70°C in batch mode in the presence (A) and absence (B) of *C. saccharolyticus *cell-free growth supernatant. Symbols: "○", biomass (*OD*_620_); "●", accumulative H_2_. The experiment is a representative of two independent replicates.

The outcome of the *in silico *simulations provoked our interest to confirm the presence of any secretory products of *C. *saccharolyticus that could enhance the growth of *C. kristjanssonii*. For this, a total inoculum volume of *C. kristjanssonii *of 15% (v/v), in which one third of the liquid phase was replaced by *C. saccharolyticus *cell-free, spent-culture broth, was used to initiate a batch culture on glucose (10 g L^-1^). As illustrated in Figure 6, the pure culture of *C. kristjanssonii *supplemented with *C. saccharolyticus *cell-free growth supernatant had, indeed, a shorter lag phase than a control culture that lacked *C. saccharolyticus *culture supernatant. Moreover, the treated culture exhibited around 18% higher maximum biomass concentration, which corresponds to an increase in CDW by 0.16 g L^-1^. No significant change in the maximum specific growth rate of *C. kristjanssonii*, however, was observed in the treated culture. To further amplify the possible growth-enhancing effect, a lower inoculum volume of *C. kristjanssonii *was used, i.e., 10%, in which 50% of the liquid phase was replaced by *C. saccharolyticus *cell-free growth supernatant. Under this condition, the treated culture exhibited a lag phase of only 5 h, compared to 54 h in the control (data not shown).

## Discussion

Hydrogen-producing co-cultures constructed *de novo *may offer a better performance than mixed-population enrichments, while overcoming some of the limitations of pure cultures based on synergies among the microorganisms involved. In several previous studies on defined co-cultures, it was a common strategy to proceed by isolating the dominant members of a mixed enrichment culture and then recombining the isolates in order to reproduce the stability and function of the original microflora [17,21,29,30]. Here, we combined two closely-related strains from geographically distant, albeit similar, habitats, i.e., *C. saccharolyticus *DSM 8903 and *C. kristjanssonii *DSM 12137. The former bacterium was isolated from a thermal pool in New Zealand [31,32], whereas the latter was isolated from a biomat sample of an Icelandic hot spring [33]. Both organisms are strictly anaerobic, extreme thermophilic heterotrophs with the ability to degrade complex polysaccharides and ferment both hexose and pentose sugars [28,31,33,34].

A CSTR seeded with *C. saccharolyticus *and *C. kristjanssonii *could be successfully operated over a period of 70 days at different dilution rates with both species coexisting in the system, when glucose and xylose (10 g L^-1^) were used as the main carbon and energy sources. According to steady-state residual sugar analysis, the culture was carbon-sufficient at all dilution rates, indicating that another nutrient must have been limiting the growth. Based on the standard biomass formula, i.e., CH_1.8_O_0.5_N_0.2 _[35], and the maximum biomass concentration obtained by either organism in batch fermentations (ca. 1 gCDW L^-1^), the amount of inorganic nitrogen in the feed, present as NH_4_Cl, is considered to be in excess. A component in yeast extract (YE), e.g., a growth factor, is most likely to be responsible for growth limitation and incomplete sugar utilization by the co-culture, since the YE-to-sugar ratio in the feed was only 1/10. This kind of limitation has been previously suggested for *C. saccharolyticus *[36] and for *Thermoanaerobacter ethanolicus *[37] during continuous fermentation of glucose and xylose, respectively. In our study, this was verified by increasing the YE-to-sugar ratio in the feed to 1/4, where the co-culture could be carbon limited when grown on a mixture of glucose and xylose (Table 3) or glucose only (Table 4).

In general, the H_2 _yields obtained in *C. saccharolyticus*-*C. kristjanssonii *co-cultures in this study under both carbon and non-carbon limited conditions are significantly higher than the yields previously reported for defined co-cultures [29,38-40] or mixed-culture enrichments [3,23,41], regardless of the type of reactor employed. The decrease in H_2 _yield with the dilution rate observed in the co-culture fermentations as well as in the pure cultures of *C. saccharolyticus *and *C. kristjanssonii *was, in part, a result of increased lactate formation (see Additional file 2), which drains some of the electrons required for H_2 _production. The highest H_2 _yield of the co-culture was 3.7 mol per mol of hexose sugar (i.e., 92.5% of the maximum theoretical H_2 _yield that can be achieved via dark fermentation). This yield could only be achieved by limiting the co-culture on glucose at low *D *(i.e., 0.06 h^-1^) and was higher than that obtained by either organism in pure culture under the same experimental conditions (Table 4), alluding to a possible synergistic effect of the two strains on H_2 _production. This also indicates that carbon limitation can be a successful strategy for improving the sugar-conversion efficiency and increasing H_2 _yield of the co-culture. Although it remains unclear why limiting the cells for glucose leads to what appears to be more efficient substrate utilization and increased H_2 _production, Bisaillon et al [42] suggested the possibility of carbon flow to secondary metabolic pathways, for example, glycogen synthesis, being restricted at low glucose concentrations thereby shunting most of the carbon to the catabolic, H_2_-generating, pathways.

In contrast to H_2 _yield, the volumetric H_2 _production rate of the co-culture constructed in this study increased by increasing the dilution rate, which agrees with data from previous reports on fermentative H_2 _production in chemostat cultures [36,43]. The maximum H_2 _production rate achieved by the co-culture at the highest *D *tested in this study (Table 2) is equivalent to 0.28 L_H2 _L_culture_^-1 ^h^-1^, which lies within the upper range of previously reported productivities of mixed culture-fermentations in CSTRs [41,44-46]. The optimum balance between yield and productivity was observed under glucose limitation at steady state of the higher *D *(Table 4), implying that both carbon limitation and the dilution rate are critical factors for optimizing H_2_-production efficiency by the co-culture. Under those conditions, the volumetric H_2 _production rate of the co-culture was equivalent to that of *C. kristjanssonii*, which indeed was the predominant organism in the co-culture. H_2 _yield, however, was significantly higher than that of *C. kristjanssonii*, pointing again at a possible synergistic effect of the two strains on H_2 _production.

An interesting outcome of the present study is the ability of the two closely related *Caldicellulosiruptor *species to stably coexist over the whole range of conditions tested. Since both species occupy the same trophic level [31,33], they are expected to compete for the same growth-limiting substrate in a chemostat culture. Based on the competitive exclusion principle [47], this competition should result in one strain in the co-culture completely displacing the other in the development towards a steady state under a given condition. Since YE, which is a complex nutrient, was suspected for growth limitation in the co-culture under carbon-sufficient conditions, the coexistence could be explained in that each species was limited on a different component in YE. However, the stable coexistence of the two strains observed at steady-state conditions with glucose as the sole growth-limiting substrate cannot be explained on that basis. As discussed below, the reproducible pattern of population dynamics observed under different conditions in the present study provides a clue on the occurrence of interspecies interaction in the co-culture, which could be the reason behind the stable coexistence of *C. saccharolyticus *and *C. kristjanssonii*.

The prevalence of an organism in a batch culture depends on its maximum specific growth rate compared with that of the other organisms capable of growth in the inoculum [2]. Since *C. kristjanssonii *exhibits a 40% lower maximum specific growth rate than that of *C. saccharolyticus *during batch growth on glucose and xylose [28], the prevalence of the former in their co-culture during the batch start-up phase and at higher dilution rates implies that it acquires a higher specific growth rate in presence of the latter. In addition, the biomass yield of the *C. kristjanssonii*-dominated co-culture in glucose-limited chemostat cultivations at a *D *of 0.15 h^-1 ^was significantly higher than that of *C. kristjanssonii *alone under otherwise the same conditions (Table 4), which points to an enhancing effect of *C. saccharolyticus *on the biomass yield of *C. kristjanssonii*. Indeed, the fact that none of the strains was completely displaced by the other at any of the steady states evaluated supports the hypothesis of the occurrence of a beneficial sort of interspecies interaction in the co-culture rather than only competition for the same growth-limiting nutrient. The simple mathematical model we developed to describe this proposed interaction, in terms of compound *E *that has a growth-enhancing effect on *C. kristjanssonii*, could successfully mimic the stable coexistence and predict the population dynamics in the co-culture, ruling out any other required mechanisms (Figure 5). Furthermore, it is the combination of enhancing the growth rate and biomass yield of *C. kristjanssonii *that creates the condition for stable co-existence as observed in the chemostat-cultivations. This condition only remains valid as long as *C. saccharomyces *stays in the system to provide the proposed growth-enhancing compound. The reduction in the lag phase and the increase in maximum biomass concentration of *C. kristjanssonii *in the presence of *C. saccharolyticus *growth supernatant (Figure 6) provided experimental evidence on the presence of such a compound or signaling molecule that can enhance the growth of *C. kristjanssonii*. Although the maximum specific growth rate was not affected under these conditions, this could be due to the absence of *C. saccharolyticus *cells in the reactor, thus preventing the *continuous *supply of the growth-enhancing compound. It cannot also be ruled out that this compound is actually a product of cell lysis rather than a secretory product since *C. saccharolyticus *cells are prone to lyse significantly [48,49].

In light of the social evolution theory of microorganisms [50], the ability of *C. saccharolyticus *to enhance the growth of *C. kristjanssonii *might be classified as an altruistic cooperation. An explanation for altruistic cooperation between close relatives is best provided by the kin selection theory, where an individual species can still pass on its own genes to the next generation indirectly by helping a closely related species to reproduce. Unraveling the precise mechanism of this interaction or the nature of the involved molecules, however, remains a conceptual and methodological challenge.

It is well known that each population or individual detects and responds to the presence of others in a consortium by trading metabolites or by exchanging dedicated molecular signals [1,51]. Growth enhancement as a consequence of interspecies interactions in bacteria may occur in response to some signaling molecules [52]. Processed oligopeptides are known quorum sensing molecules, or autoinducers, that are used by Gram-positive bacteria for communication [53]. Nichols et al [54] presented evidence that short peptides may be essential factors for initiating growth of 'uncultivable' cells. The existence of a peptide-based quorum sensing in hyperthermophilic bacteria was also previously demonstrated in a co-culture of *Thermotoga maritima *and *Methanococcus jannaschii *and was responsible for inducing exopolysaccharide production and enhancing biofilm formation by the former organism [13]. Moreover, it has been recently suggested that the addition of a second microbe, viz. *Tm. maritima*, to a pure culture of *C. saccharolyticus *triggers events causing the presence, absence and differential expression of protein species within the system [15]. Although the genome of *C. saccharolyticus *has been fully sequenced and annotated [34], it is difficult to examine for the presence of putative small peptide signaling molecules since signaling peptides are often products of genes encoding proteins less than 100 amino acids in length. In *C. saccharolyticus *genome, there are 496 of such genes, around 55% of which encode for hypothetical proteins (NCBI Entrez; http://www.ncbi.nlm.nih.gov/sites/entrez).

While genomic information did not prove useful, other approaches, such as microarray-based functional genomics approaches [12,14] or bioassay-directed fractionation and analysis of the growth supernatant [54], can be adopted for the identification of candidate signaling molecules and understanding the molecular mechanisms behind the interactions in the co-culture. This is, however, beyond the scope of the current study and may become more feasible by the availability of the complete genome sequence of *C. kristjanssonii*.

## Conclusions

The present study provides essential evidence on the possibility of stable co-existence of two closely related bacteria isolated from distant habitats in a continuous-flow system under steady-state conditions. This augments the suggestion of *de novo *constructed or 'designer' co-cultures as potential alternatives for several biotechnological applications that are, otherwise, carried out using mixed culture enrichments. Although the increase in H_2 _yield by the co-culture constructed in this study was not dramatic, as compared with that of the individual strains, it is still higher than the H_2 _yield reported for any mixed culture to date. Generally, benefits of the use of the co-culture other than improving product yield may include enhanced resistance to invasion by other species and increased chance of biofilm formation [55]; the latter being a desirable feature in several industrial fermentation systems. Extending the range of substrate utilization is another advantage that can be gained by combining *C. saccharolyticus *and *C. kristjanssonii *in a co-culture. For example, *C. saccharolyticus *ferments L-arabinose and L-rhamnose, whereas *C. kristjanssonii *does not grow on these sugars [33] and probably the latter would be able to utilize some substrates not readily consumed by the former, which merits further investigation.

## Competing interests

The authors declare that they have no competing interests.

## Authors' contributions

AAZ conceived of the study, designed and performed the experimental and computational work and drafted the manuscript. PR participated in the design of the PCR assays and critically revised the manuscript. EWJN developed the mathematical model, participated in interpreting the experimental results and critically revised the manuscript. All authors read and approved the final manuscript.

## Supplementary Material

Additional file 1 **Values of different parameters included in the co-culture model**. Click here for file

Additional file 2 **Fermentation details of different chemostat cultivations**. Product yields, specific rates of substrate consumption and metabolite formation and carbon and electron recoveries in different chemostat cultivations of *C. saccharolyticus*, *C. kristjanssonii *and/or their co-culture (8 tables). Click here for file

## References

[B1] Brenner K You LC Arnold FH Engineering microbial consortia: a new frontier in synthetic biology Trends Biotechnol 2008 26 483 489 10.1016/j.tibtech.2008.05.004 18675483

[B2] Stanbury PF Whitaker A Hall SJ Principles of Fermentation Technology 2000 2 Butterworth Heinemann, Oxford

[B3] Kleerebezem R van Loosdrecht MCM Mixed culture biotechnology for bioenergy production Curr Opin Biotechnol 2007 18 207 212 10.1016/j.copbio.2007.05.001 17509864

[B4] Weibel DB Building communities one bacterium at a time Proc Natl Acad Sci USA 2008 105 18075 18076 10.1073/pnas.0810201106 19020083 PMC2587591

[B5] Sieuwerts S de Bok FA Hugenholtz J van Hylckama Vlieg JE Unraveling microbial interactions in food fermentations: from classical to genomics approaches Appl Environ Microbiol 2008 74 4997 5007 10.1128/AEM.00113-08 18567682 PMC2519258

[B6] Pfeiler EA Klaenhammer TR The genomics of lactic acid bacteria Trends Microbiol 2007 15 546 553 10.1016/j.tim.2007.09.010 18024129

[B7] Fu N Peiris P Markham J Bavor J A novel co-culture process with *Zymomonas mobilis *and *Pichia stipitis *for efficient ethanol production on glucose/xylose mixtures Enz Microb Technol 2009 45 210 217 10.1016/j.enzmictec.2009.04.006

[B8] Qian MY Tian S Li XF Zhang J Pan YP Yang XS Ethanol production from dilute-acid softwood hydrolysate by co-culture Appl Biochem Biotechnol 2006 134 273 283 10.1385/ABAB:134:3:273 16960285

[B9] Mamma D Koullas D Fountoukidis G Kekos D Macris BJ Koukios E Bioethanol from sweet sorghum: Simultaneous saccharification and fermentation of carbohydrates by a mixed microbial culture Process Biochem 1996 31 377 381 10.1016/0032-9592(95)00075-5

[B10] Collet C Schwitzguebel JP Peringer P Improvement of acetate production from lactose by growing *Clostridium thermolacticum *in mixed batch culture J Appl Microbiol 2003 95 824 831 10.1046/j.1365-2672.2003.02060.x 12969297

[B11] Taniguchi M Tokunaga T Horiuchi K Hoshino K Sakai K Tanaka T Production of L-lactic acid from a mixture of xylose and glucose by co-cultivation of lactic acid bacteria Appl Microbiol Biotechnol 2004 66 160 165 10.1007/s00253-004-1671-x 15558273

[B12] Johnson MR Conners SB Montero CI Chou CJ Shockley KR Kelly RM The *Thermotoga maritima *phenotype is impacted by syntrophic interaction with *Methanococcus jannaschii *in hyperthermophilic coculture Appl Environ Microbiol 2006 72 811 818 10.1128/AEM.72.1.811-818.2006 16391122 PMC1352257

[B13] Johnson MR Montero CI Conners SB Shockley KR Bridger SL Kelly RM Population density-dependent regulation of exopolysaccharide formation in the hyperthermophilic bacterium *Thermotoga maritima* Mol Microbiol 2005 55 664 674 10.1111/j.1365-2958.2004.04419.x 15660994

[B14] Maligoy M Mercade M Cocaign-Bousquet M Loubiere P Transcriptome analysis of *Lactococcus lactis *in coculture with *Saccharomyces cerevisiae* Appl Environ Microbiol 2008 74 485 494 10.1128/AEM.01531-07 17993564 PMC2223240

[B15] Muddiman D Andrews G Lewis D Notey J Kelly R Part II: defining and quantifying individual and co-cultured intracellular proteomes of two thermophilic microorganisms by GeLC-MS^2 ^and spectral counting Anal Bioanal Chem 2010 398 391 404 10.1007/s00216-010-3929-8 20582400 PMC3727164

[B16] Stolyar S Van Dien S Hillesland KL Pinel N Lie TJ Leigh JA Stahl DA Metabolic modeling of a mutualistic microbial community Mol Syst Biol 2007 3 92 10.1038/msb4100131 17353934 PMC1847946

[B17] Kato S Haruta S Cui ZJ Ishii M Igarashi Y Stable coexistence of five bacterial strains as a cellulose-degrading community Appl Environ Microbiol 2005 71 7099 7106 10.1128/AEM.71.11.7099-7106.2005 16269746 PMC1287685

[B18] Collet C Gaudard O Peringer P Schwitzguebel JP Acetate production from lactose by *Clostridium thermolacticum *and hydrogen-scavenging microorganisms in continuous culture - Effect of hydrogen partial pressure J Biotechnol 2005 118 328 338 10.1016/j.jbiotec.2005.05.011 15992956

[B19] Drzyzga O Gerritse J Dijk JA Elissen H Gottschal JC Coexistence of a sulphate-reducing *Desulfovibrio *species and the dehalorespiring *Desulfitobacterium frappieri *TCE1 in defined chemostat cultures grown with various combinations of sulphate and tetrachloroethene Environ Microbiol 2001 3 92 99 10.1046/j.1462-2920.2001.00157.x 11321548

[B20] Kato S Haruta S Cui ZJ Ishii M Igarashi Y Network relationships of bacteria in a stable mixed culture Microb Ecol 2008 56 403 411 10.1007/s00248-007-9357-4 18196313

[B21] Thomas S Sarfaraz S Mishra LC Iyengar L Degradation of phenol and phenolic compounds by a defined denitrifying bacterial culture World J Microbiol Biotechnol 2002 18 57 63 10.1023/A:1013947722911

[B22] Hallenbeck PC Ghosh D Advances in fermentative biohydrogen production: the way forward? Trends Biotechnol 2009 27 287 297 10.1016/j.tibtech.2009.02.004 19329204

[B23] Hawkes FR Hussy I Kyazze G Dinsdale R Hawkes DL Continuous dark fermentative hydrogen production by mesophilic microflora: Principles and progress Int J Hydrogen Energy 2007 32 172 184 10.1016/j.ijhydene.2006.08.014

[B24] Li CL Fang HHP Fermentative hydrogen production from wastewater and solid wastes by mixed cultures Crit Rev Env Sci Tec 2007 37 1 39 10.1080/10643380600729071

[B25] Valdez-Vazquez I Poggi-Varaldo HM Hydrogen production by fermentative consortia Ren Sust Energy Rev 2009 13 1000 1013 10.1016/j.rser.2008.03.003

[B26] Hallenbeck PC Fundamentals of the fermentative production of hydrogen Water Sci Technol 2005 52 21 29 16180405

[B27] Thauer RK Jungermann K Decker K Energy conservation in chemotropic anaerobic bacteria Bacteriol Rev 1977 41 100 180 860983 10.1128/br.41.1.100-180.1977 PMC413997

[B28] Zeidan AA van Niel EWJ Developing a thermophilic hydrogen-producing co-culture for efficient utilization of mixed sugars Int J Hydrogen Energy 2009 34 4524 4528 10.1016/j.ijhydene.2008.07.092

[B29] Liu Y Yu P Song X Qu YB Hydrogen production from cellulose by co-culture of *Clostridium thermocellum *JN4 and *Thermoanaerobacterium thermosaccharolyticum *GD17 Int J Hydrogen Energy 2008 33 2927 2933 10.1016/j.ijhydene.2008.04.004

[B30] Mori Y Characterization of a symbiotic coculture of *Clostridium thermohydrosulfuricum *YM3 and *Clostridium thermocellum *YM4 Appl Environ Microbiol 1990 56 37 42 16348106 10.1128/aem.56.1.37-42.1990 PMC183247

[B31] Rainey FA Donnison AM Janssen PH Saul D Rodrigo A Bergquist PL Daniel RM Stackebrandt E Morgan HW Description of *Caldicellulosiruptor saccharolyticus *gen. nov., sp. nov.: An obligately anaerobic, extremely thermophilic, cellulolytic bacterium FEMS Microbiol Lett 1994 120 263 266 10.1111/j.1574-6968.1994.tb07043.x 8076802

[B32] Sissons CH Sharrock KR Daniel RM Morgan HW Isolation of cellulolytic anaerobic extreme thermophiles from New Zealand thermal sites Appl Environ Microbiol 1987 53 832 838 16347327 10.1128/aem.53.4.832-838.1987 PMC203765

[B33] Bredholt S Sonne-Hansen J Nielsen P Mathrani IM Ahring BK *Caldicellulosiruptor kristjanssonii *sp nov., a cellulolytic extremely thermophilic, anaerobic bacterium Int J Syst Bacteriol 1999 49 991 996 10.1099/00207713-49-3-991 10425755

[B34] van de Werken HJ Verhaart MR VanFossen AL Willquist K Lewis DL Nichols JD Goorissen HP Mongodin EF Nelson KE van Niel EWJ Stams AJM Ward DE de Vos WM van der Oost J Kelly RM Kengen SWM Hydrogenomics of the extremely thermophilic bacterium *Caldicellulosiruptor saccharolyticus* Appl Environ Microbiol 2008 74 6720 6729 10.1128/AEM.00968-08 18776029 PMC2576683

[B35] Roels JA Energetics and Kinetics in Biotechnology 1983 Amsterdam: Elsevier

[B36] de Vrije T Mars AE Budde MAW Lai MH Dijkema C de Waard P Claassen PAM Glycolytic pathway and hydrogen yield studies of the extreme thermophile *Caldicellulosiruptor saccharolyticus* Appl Microbiol Biotechnol 2007 74 1358 1367 10.1007/s00253-006-0783-x 17216445

[B37] Hild HM Stuckey DC Leak DJ Effect of nutrient limitation on product formation during continuous fermentation of xylose with *Thermoanaerobacter ethanolicus *JW200 Fe(7) Appl Microbiol Biotechnol 2003 60 679 686 12664146 10.1007/s00253-002-1175-5

[B38] Lu WY Wen JP Chen Y Sun B Jia XQ Liu MH Caiyin Q Synergistic effect of *Candida maltosa *HY-35 and *Enterobacter aerogenes *W-23 on hydrogen production Int J Hydrogen Energy 2007 32 1059 1066 10.1016/j.ijhydene.2006.07.010

[B39] Wang AJ Ren NQ Shi YG Lee DJ Bioaugmented hydrogen production from microcrystalline cellulose using co-culture - *Clostridium acetobutylicum *X_9 _and *Ethanoigenens harbinense *B_49_ Int J Hydrogen Energy 2008 33 912 917 10.1016/j.ijhydene.2007.10.017

[B40] Yokoi H Tokushige T Hirose J Hayashi S Takasaki Y H_2 _production from starch by a mixed culture of *Clostridium butyricum *and *Enterobacter aerogenes* Biotechnol Lett 1998 20 143 147 10.1023/A:1005372323248

[B41] Kim SH Han SK Shin HS Effect of substrate concentration on hydrogen production and 16S rDNA-based analysis of the microbial community in a continuous fermenter Process Biochem 2006 41 199 207 10.1016/j.procbio.2005.06.013

[B42] Bisaillon A Turcot J Hallenbeck PC The effect of nutrient limitation on hydrogen production by batch cultures of *Escherichia coli* Int J Hydrogen Energy 2006 31 1504 1508 10.1016/j.ijhydene.2006.06.016

[B43] Turcot J Bisaillon A Hallenbeck PC Hydrogen production by continuous cultures of *Escherchia coli *under different nutrient regimes Int J Hydrogen Energy 2008 33 1465 1470 10.1016/j.ijhydene.2007.09.034

[B44] Gavala HN Skiadas LV Ahring BK Biological hydrogen production in suspended and attached growth anaerobic reactor systems Int J Hydrogen Energy 2006 31 1164 1175 10.1016/j.ijhydene.2005.09.009

[B45] Kongjan P Min B Angelidaki I Biohydrogen production from xylose at extreme thermophilic temperatures (70°C) by mixed culture fermentation Water Res 2009 43 1414 1424 10.1016/j.watres.2008.12.016 19147170

[B46] Koskinen PEP Lay CH Puhakka JA Lin PJ Wu SY Örlygsson J Lin CY High-efficiency hydrogen production by an anaerobic, thermophilic enrichment culture from an Icelandic hot spring Biotechnol Bioeng 2008 101 665 678 10.1002/bit.21948 18814296

[B47] Hardin G The competitive exclusion principle Science 1960 131 1292 1297 10.1126/science.131.3409.1292 14399717

[B48] van Niel EWJ Claassen PAM Stams AJM Substrate and product inhibition of hydrogen production by the extreme thermophile, *Caldicellulosiruptor saccharolyticus* Biotechnol Bioeng 2003 81 255 262 10.1002/bit.10463 12474247

[B49] Willquist K Claassen PAM van Niel EWJ Evaluation of the influence of CO_2 _on hydrogen production by *Caldicellulosiruptor saccharolyticus* Int J Hydrogen Energy 2009 34 4718 4726 10.1016/j.ijhydene.2009.03.056

[B50] West SA Griffin AS Gardner A Diggle SP Social evolution theory for microorganisms Nat Rev Microbiol 2006 4 597 607 10.1038/nrmicro1461 16845430

[B51] Keller L Surette MG Communication in bacteria: an ecological and evolutionary perspective Nat Rev Microbiol 2006 4 249 258 10.1038/nrmicro1383 16501584

[B52] Shank EA Kolter R New developments in microbial interspecies signaling Curr Opin Microbiol 2009 12 205 214 10.1016/j.mib.2009.01.003 19251475 PMC2709175

[B53] Miller MB Bassler BL Quorum sensing in bacteria Annu Rev Microbiol 2001 55 165 199 10.1146/annurev.micro.55.1.165 11544353

[B54] Nichols D Lewis K Orjala J Mo S Ortenberg R O'Connor P Zhao C Vouros P Kaeberlein T Epstein SS Short peptide induces an "uncultivable" microorganism to grow in vitro Appl Environ Microbiol 2008 74 4889 4897 10.1128/AEM.00393-08 18515474 PMC2519364

[B55] Burmølle M Webb JS Rao D Hansen LH Sørensen SJ Kjelleberg S Enhanced biofilm formation and increased resistance to antimicrobial agents and bacterial invasion are caused by synergistic interactions in multispecies biofilms Appl Environ Microbiol 2006 72 3916 3923 16751497 10.1128/AEM.03022-05 PMC1489630

