# Supplementary material for: Stable coexistence of two Caldicellulosiruptor species in a de novo constructed hydrogen-producing co-culture
Source: Microb Cell Fact. 2010 Dec 30;9:102. doi: 10.1186/1475-2859-9-102 (PMC3022713; doi:10.1186/1475-2859-9-102)
Supplement: Additional file 1 — Values of different parameters included in the co-culture model. [file 1475-2859-9-102-S1.PDF]

Table S1. Values of the parameters used in the co-culture model described in the Material & Methods section.

Parameter	Value	Unit	Reference
E_C	0.6	mmol L^{-1}	N/A
K_{s_1}	1	mmol L^{-1}	N/A
K_{s_2}	4	mmol L^{-1}	N/A
s_0	22.2	mmol L^{-1}	This study
Y_{sx_1}	0.019	gCDW $mmol^{-1}$	This study
$Y_{sx_2}^0$	0.013	gCDW $mmol^{-1}$	This study
$Y_{sx_2}^{'}$	0.026	gCDW $mmol^{-1}$	This study
κ	10	mmol $gCDW^{-1}$	N/A
$\mu_{\max 1}$	0.24	h^{-1}	This study
$\mu_{\max 2}^0$	0.14	h^{-1}	Zeidan and van Niel, 2009[a]
$\mu_{\max 2}^{'}$	0.24	h^{-1}	Zeidan and van Niel, 2009[a]

N/A = Not available.

No values were available for the affinity constants for glucose (K_{s_1} and K_{s_2}) and the parameters related to the growth-enhancing compound (κ and E_C). However, the predominance of *C. saccharolyticus* at the lower *D* observed experimentally in the current study indicates that it should have a higher affinity for glucose than *C. kristjanssonii* (i.e., $K_{s_1} < K_{s_2}$; Fig. 3). Therefore, values for these parameters were assigned via educated guess and have not been determined experimentally. Accordingly, they should not be regarded as 'true' values.

[a] Zeidan AA, van Niel EWJ: **Developing a thermophilic hydrogen-producing co-culture for efficient utilization of mixed sugars.** *Int J Hydrogen Energy* 2009, **34:**4524-4528.
