# Supplementary material for: Stable coexistence of two Caldicellulosiruptor species in a de novo constructed hydrogen-producing co-culture
Source: Microb Cell Fact. 2010 Dec 30;9:102. doi: 10.1186/1475-2859-9-102 (PMC3022713; doi:10.1186/1475-2859-9-102)
Supplement: Additional file 2 — Fermentation details of different chemostat cultivations. Product yields, specific rates of substrate consumption and metabolite formation and carbon and electron recoveries in different chemostat cultivations of C. saccharolyticus, C. kristjanssonii and/or their co-culture (8 tables). [file 1475-2859-9-102-S2.PDF]

Table S2.1. Specific rates of sugar consumption and product formation by the co-culture at steady states of different dilution rates during co-fermentation of glucose and xylose (10 g L^{-1}; 1:1) at pH 6.7 and 70 °C.

D (h^{-1})	Specific consumption/production rate (mmol $gCDW^{-1}$ h^{-1})					
	$q_{glucose}$	q_{xylose}	q_{H2}	q_{CO2}	$q_{acetate}$	$q_{lactate}$
0.04	1.0	3.1	12.7	6.6	5.9	0.1
0.06	1.1	3.6	14.2	7.5	6.6	0.6
0.08	1.0	4.1	15.5	8.9	7.2	0.4
0.12	1.2	4.5	15.0	7.7	7.5	0.6
0.15	1.2	5.1	18.4	9.2	9.0	1.4
0.2	1.7	5.9	18.3	9.3	9.1	0.8
0.25	1.6	6.9	21.0	10.8	9.8	1.3
0.3	2.1	7.6	21.0	10.8	11.2	1.0

Table S2.2. Product yields at steady states of different dilution rates during co-fermentation of glucose and xylose (10 g L^{-1}; 1:1) by the co-culture at pH 6.7 and 70 °C.

D (h^{-1})	Product yield [mol (mol C6)$^{-1}$]				Carbon balance	Redox balance
	Y_{H2}	Y_{CO2}	$Y_{acetate}$	$Y_{lactate}$		
0.04	3.5	1.8	1.6	0.02	0.93	0.93
0.06	3.6	1.9	1.6	0.15	1.04	1.02
0.08	3.5	1.8	1.6	0.08	1.01	1.00
0.12	3.1	1.6	1.5	0.13	1.00	1.00
0.15	2.9	1.4	1.4	0.20	0.98	0.99
0.2	2.8	1.4	1.4	0.12	0.98	0.98
0.25	2.9	1.5	1.4	0.18	1.02	1.03
0.3	2.5	1.3	1.4	0.12	0.97	0.98

Table S3.1. Specific rates of sugar consumption and product formation by the co-culture at steady states of different dilution rates during co-fermentation of glucose and xylose (4 g L^{-1}; 1:1) at pH 6.7 and 70 °C. Values are means of two independent replicates ± standard deviation.

D (h^{-1})	Specific consumption/production rate (mmol $gCDW^{-1}$ h^{-1})					
	$q_{glucose}$	q_{xylose}	q_{H2}	q_{CO2}	$q_{acetate}$	$q_{lactate}$
0.06	1.4±0.13	1.8±0	10.2±0.3	5.6±0.4	5.1±0.5	0.06±0
0.15	3.6±0.45	4.9±0.05	24.7±1.13	13.3±0.8	12.0±1.5	0.07±0.02

Table S3.2. Product yields at steady states of different dilution rates during co-fermentation of glucose and xylose (4 g L^{-1}; 1:1) by the co-culture at pH 6.7 and 70 °C. Values are means of two independent replicates ± standard deviation.

D (h^{-1})	Product yield [mol (mol C6)$^{-1}$]				Carbon balance	Redox balance
	Y_{H2}	Y_{CO2}	$Y_{acetate}$	$Y_{lactate}$		
0.06	3.6±0.04	2.0±0.5	1.8±0.1	0.02±0	1.07±0.04	1.05±0.02
0.15	3.2±0.02	1.7±0.01	1.5±0.1	0.01±0	0.95±0.03	0.93±0.03

Table S4.1. Specific rates of sugar consumption and product formation by *C. saccharolyticus*, *C. kristjanssonii* and their co-culture at steady states of different dilution rates on glucose (4 g L^{-1}) at pH 6.7 and 70 °C. Values are means of two independent replicates ± standard deviation.

Organism(s)	*D* (h^{-1})	Specific consumption/production rate (mmol $gCDW^{-1}$ h^{-1})				
		$q_{glucose}$	q_{H2}	q_{CO2}	$q_{acetate}$	$q_{lactate}$
C. saccharolyticus	0.06	3.8±0.04	13.2±0.08	7.1±0.13	6.3±0.3	0.06±0
	0.15	7.8±0.4	24.4±1	13.1±1	11.7±0.2	0.06±0
C. kristjanssonii	0.06	5.1±0.02	17.8±0.5	9.5±0.5	8.7±0.4	0.15±0.03
	0.15	11.6±1	34.6±1.5	18.4±0.7	16.7±0.4	2.3±0.2
Co-culture	0.06	4.0±0.2	14.8±0.8	7.9±0.4	7.0±0.9	0.1±0.02
	0.15	6.1±0.2	21.4±0.8	11.4±0.3	9.6±0.6	0.79±0.02

Table S4.2. Product yields of *C. saccharolyticus*, *C. kristjanssonii* and their co-culture at steady states of different dilution rates on glucose (4 g L^{-1}) at pH 6.7 and 70 °C. Values are means of two independent replicates ± standard deviation.

Organism(s)	*D* (h^{-1})	Product yield [mol (mol C6)$^{-1}$]				Carbon balance	Redox balance
		Y_{H2}	Y_{CO2}	$Y_{acetate}$	$Y_{lactate}$		
C. saccharolyticus	0.06	3.5±0.1	1.9±0.05	1.7±0.07	0.01±0	0.98±0.01	0.96±0.02
	0.15	3.1±0.2	1.7±0.05	1.5±0.05	0.01±0	0.92±0.02	0.91±0.03
C. kristjanssonii	0.06	3.5±0.1	1.9±0.1	1.7±0.11	0.03±0	0.97±0.01	0.95±0.02
	0.15	3.0±0.1	1.6±0.05	1.5±0.03	0.21±0.02	0.93±0.03	0.92±0.03
Co-culture	0.06	3.7±0	2.0±0	1.8±0.14	0.03±0	1.04±0.04	1.02±0.04
	0.15	3.5±0	1.9±0.02	1.6±0.05	0.13±0	1.06±0.01	1.05±0.02

Table S5.1. Specific rates of sugar consumption and product formation by the co-culture at steady states of different dilution rates on xylose (4 g L^{-1}) at pH 6.7 and 70 °C. Values are means of two independent replicates ± standard deviation.

D (h^{-1})	Specific consumption/production rate (mmol $gCDW^{-1}$ h^{-1})				
	q_{xylose}	q_{H2}	q_{CO2}	$q_{acetate}$	$q_{lactate}$
0.06	10.0±2.3	22.6±4.1	12.1±2.3	13.5±1.1	0.7±0.02
0.15	14.5±4.1	33.0±8.9	17.8±4.8	18.4±1.2	1.3±0.3

TABLE S5.2. Product yields of the co-culture at steady states of different dilution rates on xylose (4 g L^{-1}) at pH 6.7 and 70 °C. Values are means of two independent replicates ± standard deviation.

D (h^{-1})	Product yield [mol (mol C6)$^{-1}$]				Carbon balance	Redox balance
	Y_{H2}	Y_{CO2}	$Y_{acetate}$	$Y_{lactate}$		
0.06	2.7±0.1	1.5±0.1	1.7±0.2	0.09±0.02	0.89±0.11	0.88±0.11
0.15	2.7±0.0	1.5±0.0	1.6±0.3	0.09±0.0	0.91±0.14	0.89±0.14
